# Hypoxia Worsens Affective Responses and Feeling of Fatigue During Prolonged Bed Rest

**DOI:** 10.3389/fpsyg.2018.00362

**Published:** 2018-03-23

**Authors:** Nektarios A. M. Stavrou, Tadej Debevec, Ola Eiken, Igor B. Mekjavic

**Affiliations:** ^1^School of Physical Education and Sport Science, National and Kapodistrian University of Athens, Athens, Greece; ^2^Department of Sport Psychology, Hellenic Sports Research Institute, Olympic Athletic Center of Athens “Spiros Louis”, Athens, Greece; ^3^Department for Automation, Biocybernetics and Robotics, Jozef Stefan Institute, Ljubljana, Slovenia; ^4^Faculty of Sport, University of Ljubljana, Ljubljana, Slovenia; ^5^Department of Environmental Physiology, Swedish Aerospace Physiology Centre, Royal Institute of Technology, Stockholm, Sweden; ^6^Department of Biomedical Physiology and Kinesiology, Simon Fraser University, Burnaby, BC, Canada

**Keywords:** bed rest, inactivity, hypoxia, affect, fatigue, motivation

## Abstract

Previous research, although limited, suggests that both hypoxia and bed rest influence psychological responses by exaggerating negative psychological responses and attenuating positive emotions. The present study investigated the effect of a 21-day prolonged exposure to normobaric hypoxia and bed rest on affective responses and fatigue. Eleven healthy participants underwent three 21-day interventions using a cross-over design: (1) normobaric hypoxic ambulatory confinement (HAMB), (2) normobaric hypoxic bed rest (HBR) and (3) normoxic bed rest (NBR). Affective and fatigue responses were investigated using the Activation Deactivation Adjective Check List, and the Multidimensional Fatigue Inventory, which were completed before (Pre), during (Day 7, Day 14, and Day 21) and after (Post) the interventions. The most negative psychological profile appeared during the HBR intervention. Specifically, tiredness, tension, general and physical fatigue significantly increased on days 7, 14, and 21, as well as at Post. After the HBR intervention, general and physical fatigue remained higher compared to Pre values. Additionally, a deterioration of psychological responses was also noted following HAMB and NBR. In particular, both hypoxia and BR *per se* induced subjective fatigue and negative affective responses. BR seems to exert a moderate negative effect on the sensation of fatigue, whereas exercise attenuates the negative effects of hypoxia as noted during the HAMB condition. In conclusion, our data suggest that the addition of hypoxia to bed rest-induced inactivity significantly worsens affective responses and feeling of fatigue.

## Introduction

It is envisaged that future long-term space explorations will establish permanent habitats on the Moon and Mars. To minimize the risk of decompression sickness (DCS) of astronauts preparing for extravehicular activities, these habitats will be hypobaric and hypoxic (Bodkin et al., 2006). Namely, the pressure inside the space suits is about one third of the normobaric pressure in the International Space Station (ISS). During the preparation for extravehicular activity (EVA), astronauts undergo a slow decompression lasting several hours, and breathe oxygen, to enhance nitrogen elimination from the body, thus minimizing the etiology of venous gas emboli, and the risk of DCS. Whereas EVAs are not frequent events on the ISS, they will undoubtedly be conducted on a daily basis during missions on the Moon and Mars. The cumulative effect of repeated decompression procedures, as currently practiced on the ISS, are therefore not optimal during Moon and Mars missions. By maintaining the ambient pressure in future habitats at the same pressure as the space suits, but with a higher fraction of oxygen, the need for decompression will be eliminated.

Exposure to microgravity initiates a process of adaptation in all physiological systems. To date, this process has been investigated in ground-based studies on Earth, simulating the effect of gravity with the bed rest (BR) experimental model. Previous work in this area has confirmed that the effects of an exposure to microgravity for a given length of time are similar to the effects observed following BR of the same duration (Pavy-Le Traon et al., 2007). Since in future Moon and Mars missions, astronauts will be exposed to both hypoxia and reduced gravity, a program of research was initiated by the European Space Agency (ESA) to investigate the interaction of reduced gravity, simulated with the BR experimental model, and hypoxia, on the process of adaptation of physiological systems, previously investigated only in normoxia. Whereas the independent effects of hypoxia and prolonged BR on the adaptation of different physiological systems are well established (Pavy-Le Traon et al., 2007; Bartsch and Saltin, 2008), the psychological responses to such interventions have, to date, received little attention (Ishizaki et al., 2002; Liu et al., 2012). In addition, previous psychological studies have predominantly focused on the separate effects of hypoxia (Hornbein et al., 1989; Virués-Ortega et al., 2004), reduced gravity (Kanas et al., 2009) or unloading (Lipnicki and Gunga, 2009).

Recently the independent and combined effects of hypoxia and BR on psychological status were examined during 10-day intervention (Stavrou et al., 2015) with the data clearly indicating a detrimental effect of hypoxia. In short, concomitant hypoxia and BR seem to augment negative psychological responses, whereas positive psychological characteristics are diminished. However, these findings suggest that hypoxic ambulatory confinement (HAMB) and normoxic BR (NBR) do not significantly alter participants’ psychological responses during or after the intervention. These results concur with the conclusions of DeRoshia and Greenleaf (1993) that BR does not deteriorate psychological responses, such as motivation, activation and concentration. In contrast, Lipnicki and Gunga (2009) reported considerable BR-induced alterations ranging from deterioration to improvement of cognitive function.

In contrast to inactivity, exercise is known to positively influence psychological responses by increasing positive and minoring negative emotions (Stavrou et al., 2015). Indeed, the positive impact of exercise on mood, affect, cognition, and brain activity is well-established (Ekkekakis, 2003; Ekkekakis et al., 2011), although the magnitude of the effect is highly dependent on exercise type/intensity as well as the participants’ characteristics (e.g., age, physical condition) (Hillman et al., 2008; Erickson et al., 2015).

Studies investigating the neuropsychological changes, in response to systemic reduction of oxygen (i.e., oxygen availability within the central nervous system under hypoxic conditions) clearly demonstrate a variety of impairments including alterations in mood, behavior, fatigue, motivation, and cognitive function (Gallagher and Hackett, 2004; Bartsch and Saltin, 2008). Other symptoms accompanying hypoxic exposure also include, impairments of coordination, cognitive function, vigor, etc. (Acevedo and Ekkekakis, 2001; Virués-Ortega et al., 2004). These symptoms are usually accompanied by elevation of negative psychological responses such as apathy, anxiety, negative mood, fatigue, confusion, hostility and a decrease of the positive psychological profile elements, such as activation and positive affect (Ishizaki et al., 2002; Lipnicki and Gunga, 2009). Hypoxia has also been linked to changes in brain activity that could importantly modulate the sensation of psychological fatigue. This has been demonstrated through electrophysiological methods, such as electroencephalography (EEG), and event-related potentials (ERPs) (Ozaki et al., 1995; Wilson et al., 2009; Richardson et al., 2011).

Among the aforementioned symptoms, and one that seems especially important during high altitude sojourns, is the increased sense of fatigue. However, to date, studies have mainly focused on the physiological aspects of fatigue, even though its psychological characteristics are of great interest and can importantly modulate individuals’ operational and/or physical capacity. Fatigue is a multifactorial concept, which has been conceptualized in a dualistic manner (acute vs. chronic, physiological vs. psychological, central vs. peripheral) characterized by emotional, behavioral, and cognitive aspects (Lewis and Wessely, 1992; Shen et al., 2006). For the purpose of the present study, we employed the conceptualization of fatigue as a self-reported state mood construct of perceived fatigability, which refers to an individual’s reduced motivation, cognitive function and capacity for accomplishing mental or physical activities, characterized by emotional strain (O’connor, 2004; Enoka and Duchateau, 2016). The role, as well as the perception, of mental fatigue has been scarcely studied in both BR studies and hypoxic environments. Nevertheless it seems established that it can significantly affect decision-making, cognitive performance, psychological responses, and physical performance (Lorist et al., 2000; Marcora et al., 2009; Van Custem et al., 2017). A recent review (Virués-Ortega et al., 2004) also suggests that fatigue might have a mediating or moderating role on psychological and cognitive responses in a hypoxic environment. Interestingly, there also seems to be a negative link between low physical activity levels and fatigue (Chen, 1986; Kroenke et al., 1988). Patients characterized by chronic fatigue syndrome, who were physically inactive expressed higher fatigue levels than those who were more active (Vercoulen et al., 1996a,b). Vercoulen et al. (1998) proposed a causal relationship between low levels of physical activity and fatigue severity which might create a vicious circle that could lead to avoiding physical activity.

The present study aimed to investigate the effects of prolonged inactivity in normobaric hypoxic environment on affective responses and perception of fatigue during a 21-day experimental protocol. The psychological and fatigue responses observed during HBR intervention were compared to responses during NBR and HAMB interventions. Based on the above, we hypothesized that the HBR condition, combining both hypoxic exposure and BR-related inactivity will provoke significantly higher levels of negative psychological responses when compared to the NBR and HAMB interventions.

## Materials and Methods

This study was part of a larger project (Planetary Habitat Simulation) initiated by the European Commission, investigating the physiological and psychological effects of hypoxia and BR in healthy humans. All experimental procedures were conducted according to the ESA BR protocol recommendations (Standardization of bed rest study conditions 1.5, August 2009) (ESA, 2009) and conformed to the principles of the Declaration of Helsinki. The study protocol was approved by the National Committee for Medical Ethics at the Ministry of Health of the Republic of Slovenia.

### Participants

Subjects were recruited according to the above-mentioned ESA Standardization of BR study conditions protocol. In addition to the exclusion criteria outlined in the standardization protocol, individuals recently (<2 months) exposed to altitudes above 2000 m were also ineligible to participate. Finally, fourteen participants were selected to participate following an extensive selection process including series of interviews, fitness tests, and medical examinations. Additionally, detailed written and oral information regarding the experimental intervention and overall methodology were provided. Participants signed written informed consents, before enrolling in the study. Due to personal reasons and medical issues, three participants did not participate in the last intervention and have been excluded from the present dataset. Consequently, eleven healthy, sea level residents [age: 27 ± 6 years (mean ± SD); stature: 180 ± 3 cm; body mass: 77 ± 12 kg; BMI: 23.7 ± 3.0 kg⋅m^-2^; body fat %: 21 ± 5%; VO_2max_: 44.3 ± 6.1 mL⋅kg^-1^⋅min^-1^) completed the three intervention periods.

### General Study Outline

Detailed methodological outline of the Planetary Habitat Simulation (PlanHab) project is reported elsewhere (Debevec et al., 2014a). Briefly, the participants underwent the following three interventions in a randomized-counterbalanced manner: (1) normobaric HAMB (partial pressure of inspired oxygen (P_i_O_2_) = 90.0 ± 0.4; ∼4000 m simulated altitude), (2) normobaric HBR (P_i_O_2_ = 90.0 ± 0.4; ∼4000 m simulated altitude), and (3) normobaric NBR (P_i_O_2_ = 133.1 ± 0.3). All experimental interventions were performed at the Olympic Sports Centre Planica (Rateče, Slovenia), situated at an altitude of 940 m. During the study, two participants per day entered each condition in a sequential and fixed order. For each participant, the interventions lasted 32 days and comprised of the following distinct phases: (1) Preliminary phase that lasted 7 days upon arrival to the facility. This phase enabled familiarization of the participants with the experimental facility, and included baseline measurements (Pre); (2) Confinement phase, comprising 21 days (Days 1–21) during which the participants were exposed to their designated condition (HAMB, HBR, and NBR) and (3) Recovery phase, that ensured cautious re-ambulation of the participants during the course of 4 days and obtainment of the post-intervention measurements (Post). A 4-month wash-out period was implemented between the interventions.

### Bed Rest and Hypoxic Procedures

Two participants were accommodated in each room and were awakened daily at 7:00 AM with lights turned off at 11:00 PM. Napping was not allowed during the day. The conditions within the facility remained stable throughout all experimental conditions (ambient temperature = 24.4 ± 0.7°C; relative humidity = 53.5 ± 5.4% and ambient pressure = 684 ± 4 mm Hg).

The participants were confined to strict horizontal BR, a ground-based simulation model of microgravity-induced physiological adaptations (Pavy-Le Traon et al., 2007), during both the HBR and NBR interventions. All habitual activities (i.e., feeding, hygiene etc.) were performed in the horizontal position. The participants were allowed to use one pillow for head support. No physical activity, apart from changing position, was permitted during the BR confinements. Compliance to the protocol was ensured using 24-h closed-circuit television monitoring and permanent medical staff supervision.

During the HAMB confinement, the participants were allowed to move freely in the common hypoxic area, were encouraged to engage in their usual daily activities and also performed two, low-intensity exercise sessions (30-min) per day to mimic their habitual levels of activity. The exercise mode (stepping, cycling, or dancing) of these sessions was changed daily to avoid monotony.

The normobaric hypoxic environment was provided and maintained using a Vacuum Pressure Swing Adsorption system (b-Cat, Tiel, Netherlands) that generated and delivered the oxygen-depleted gas to the designated rooms and the common hypoxic area. The ambient air in each room was analyzed for oxygen and carbon dioxide content at 15-min intervals using calibrated analyzers. Throughout both hypoxic experimental conditions, the participants wore portable ambient oxygen concentration analyzers (Rae PGM-1100, California, United States) as a safety precaution.

### Daily Physiological Monitoring

Heart rate (HR) and capillary oxyhemoglobin saturation (SpO_2_) were measured daily in the morning, using short-range telemetry (iBody, Wahoo Fitness, Atlanta, GA, United States) and finger oximetry device (3100 WristOx, Nonin Medical, Minnesota, United States), respectively. To determine the presence of acute mountain sickness, the participants also completed the Lake Louise acute mountain sickness questionnaire each day (Roach et al., 1993). The Lake Louise score (LLS; 0-15) was calculated and the following two criteria were used to diagnose acute mountain sickness: (1) LLS score of ≥3; (2) Headache.

### Multidimensional Fatigue Inventory

The participants’ fatigue state was measured using the Multidimensional Fatigue Inventory (MFI) (Smets et al., 1995). The MFI is a 20-item self-evaluation questionnaire containing the following five subscales: general fatigue (e.g., “I feel tired”), physical fatigue (e.g., “Physically I feel I am in a bad condition”), reduced activity (e.g., “I think I do very little in a day”), reduced motivation (e.g., “I dread having to do things”), and mental fatigue (e.g., “It takes a lot of effort to concentrate on things”). Participants’ responses on MFI items were provided based on a 5-point scale ranging from 5 representing “Yes, that is true” to 1 referring to “No, that is not true.”

### Activation Deactivation Adjective Check List

The Activation Deactivation Adjective Check List (ADACL) (Thayer, 1989) is a self-report instrument consisting of 20 items. The ADACL comprises four subscales: energy, tiredness, tension, calmness, each of which contains five items. The participants were asked to rate the extent to which they experience each of the affects that are described in the ADACL items. Participants answered each item based on a 4-point rating scale (i.e., “vv” to signify “definitely feel,” “v” to signify “feel slightly,” “?” to signify “cannot decide,” “no” to signify “definitely do not feel”). Then the scale was transformed and scored from “1” representing “definitely do not feel” to “4” “definitely feel”. Each ADACL factor’s total score, ranged from 5 to 20, and was produced by adding the 5-items’ score in each factor together, higher values representing higher energy, tiredness, tension, calmness.

The participants completed the questionnaires based on how they felt at the exact time of answering, during each experimental condition. The participants completed the MFI, and the ADACL during the baseline period, specifically 2 days before the onset of each experimental condition (Pre). Thereafter, they completed the questionnaires on the 7th (D7), 14th (D14), and 21th (D21) day of each condition and on the 1st day of recovery (Post) following each condition.

### Statistical Analysis

Two 3 (Conditions: HAMB, HBR, NBR) × 5 (Time: Pre, D7, D14, D21, Post) multivariate analysis of variance, with repeated measure on the second factor (RMANOVA) were computed for ADACL and MFI subscales, respectively. Based on the results of the RMANOVA, separate analysis of variance were performed, 3 (Conditions: HAMB, HBR, NBR) × 5 (Time: Pre, D7, D14, D21, Post), on each of the affect (ADACL) and fatigue (MFI) factors to examine the between-subject condition differences and the within-subject repeated measures of the experimental conditions. In cases where the assumptions of sphericity were not met in the within-subjects repeated measure analyses, based on the results of Mauchly’s test of sphericity, the Green-House Geisser correction and the corresponding degrees of freedom were applied for subsequent *F* statistic estimation. The partial eta square was also estimated ($\eta_{p}^{2}$), as well as, Cohen’s *d* and *d*_z_, as measures of the effect size for group mean differences (Cohen, 1988; Lakens, 2013). Bonferroni corrected *t*-tests followed any significant between and within effects in the ANOVA models testing pairwise comparisons. The sample size determination was based on our previous hypoxia and bed rest confinement experiments (Stavrou et al., 2015). The significance level was set at *p* < 0.05. The Statistical Package for Social Sciences (SPSS 21.0 Win) was used for all analyses.

## Results

### General Adaptation

The participants finished all interventions without any adverse issues. The general physiological data has been reported previously (Debevec et al., 2014a), but is summarized here for the convenience of the reader. While the resting HR remained stable across all interventions, it was significantly higher during both hypoxic conditions (HAMB: 68 ± 3 beats⋅min^-1^; HBR: 72 ± 2 beats⋅min^-1^) than during NBR (60 ± 2 beats⋅min^-1^; *p* < 0.05). In contrast, the SpO_2_ values were, significantly lower (*p* < 0.001) during both the HAMB (88 ± 1%) and HBR (88 ± 2%) than the NBR (97 ± 2%). The SpO_2_ values were higher during the later stages of confinement than during Day 1 both in HAMB and HBR (*p* < 0.05). The average daily LLS values were not significantly different between interventions (HAMB: 1.2 ± 0.4; HBR: 1.3 ± 0.6; NBR: 0.7 ± 0.3; *p* < 0.05). Acute mountain sickness was, however, diagnosed in three participants during the HAMB and five participants during the HBR experimental condition on Day 2.

### Baseline Psychological Measures

Two days before the start of the 21-day intervention, participants’ baseline responses to the MFI and ADACL subscales were examined across the three experimental conditions (HAMB, HBR, and NBR). The multiple analysis of variance results indicated no significant differences between the three experimental conditions on MFI (*F* = 0.566, *p* = 0.834, $\eta_{p}^{2}$ = 0.098), and ADACL (*F* = 1.232, *p* = 0.299, $\eta_{p}^{2}$ = 0.154) subscales.

### Multidimensional Fatigue Inventory

**FIGURE 1:**
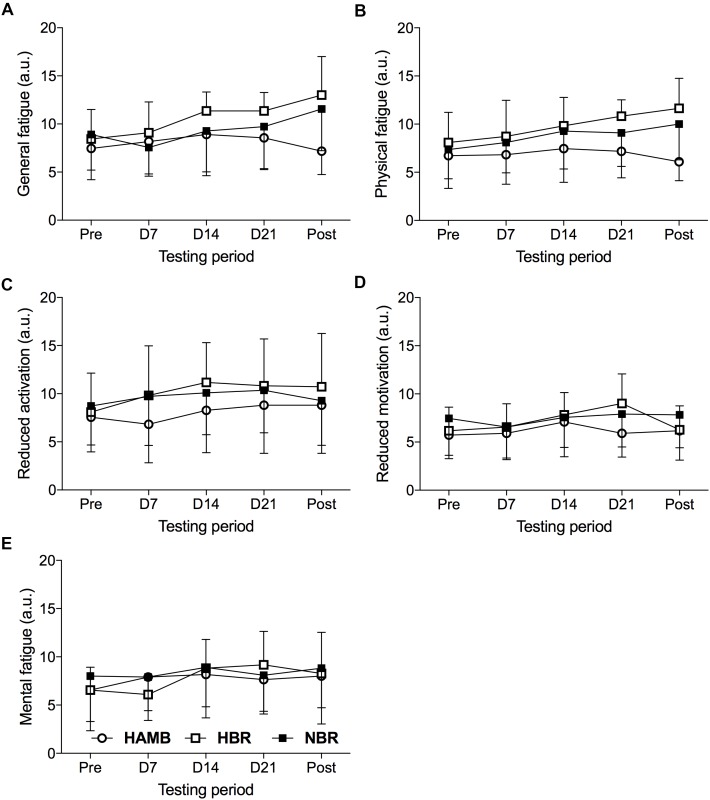
General fatigue **(A)**, physical fatigue **(B)**, reduced activation **(C)**, reduced motivation **(D)**, and mental fatigue **(E)** subscales of the Multidimensional Fatigue Inventory determined 2 days before the onset of each experimental condition (Pre), on day 7 (D7), day 14 (D14), and day 21 (D21) of each condition and 1 day following the cessation of confinement (Post) during the hypoxic ambulatory (HAMB; 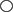), hypoxic bed rest (HBR; 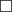), and normoxic bed rest (NBR; 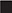) interventions (mean ± SD).

The Condition (3) × Time (5) interaction for the MFI subscales was significant (*F* = 1.947, *p* < 0.001, $\eta_{p}^{2}$ = 0.117), suggesting that factors significantly differed over time depending on the experimental condition. The means and standard deviations of the MFI subscales are presented in **Figure 1**.

The analysis of general fatigue showed a significant Condition × Time interaction (*F* = 2.569, *p* < 0.05, $\eta_{p}^{2}$ = 0.146), as well as Time (*F* = 5.956, *p* < 0.001, $\eta_{p}^{2}$ = 0.166), but not Condition (*F* = 2.421, *p* = 0.106, $\eta_{p}^{2}$ = 0.139) main effect. Examining each experimental condition, the general fatigue values in HBR (*F* = 5.190, *p* < 0.01, $\eta_{p}^{2}$ = 0.342) changed significantly over time, indicating higher values on D21 than Pre- (*p* < 0.05, *d*_z_ = 1.27) intervention measure. Additionally, differences were revealed during NBR (*F* = 4.212, *p* < 0.05, $\eta_{p}^{2}$ = 0.296). However, *post hoc* pairwise comparisons were not significant, except that an increase of general fatigue in the Post compared to the D7 measure approached statistical significance (*p* = 0.067, *d*_z_ = 1.10). Finally, no significant differences were revealed in the HAMB condition (*F* = 1.098, *p* = 0.357, $\eta_{p}^{2}$ = 0.099).

A significant Condition × Time interaction for physical fatigue (*F* = 2.481, *p* < 0.05, $\eta_{p}^{2}$ = 0.142) was detected, as well as, for Time (*F* = 6.178, *p* < 0.01, $\eta_{p}^{2}$ = 0.171) and Condition (*F* = 3.272, *p* < 0.05, $\eta_{p}^{2}$ = 0.179). The results indicate that physical fatigue increased throughout the HBR condition (*F* = 7.337, *p* < 0.001, $\eta_{p}^{2}$ = 0.423). In detail, significant differences were revealed from Pre to D21 (*p* < 0.05, *d*_z_ = 1.09), as well as from the Pre to Post measure (*p* < 0.05, *d*_z_ = 1.14), while the increased physical fatigue from D7 to D21 (*p* = 0.095, *d*_z_ = 0.88), and to the Post measures (*p* = 0.074, *d*_z_ = 0.85) approached statistical significance. In the HAMB condition, no significant changes over time appeared (*F* = 1.061, *p* = 0.355, $\eta_{p}^{2}$ = 0.096), whereas, in the NBR confinement (*F* = 2.745, *p* = 0.076, $\eta_{p}^{2}$ = 0.215) changes over time approached statistical significance. Examining the difference between the experimental conditions across the five time measures, revealed significant differences on D21 (*F* = 4.041, *p* < 0.05, $\eta_{p}^{2}$ = 0.212), during which participants’ in the HBR condition showed higher physical fatigue than in HAMB (*p* < 0.05, *d* = 1.33). In the POST measure (*F* = 3.42, *p* < 0.05, $\eta_{p}^{2}$ = 0.395), participants’ in the HBR and NBR conditions showed higher physical fatigue than in the HAMB condition (*p* < 0.001, *d* = 2.13, *p* < 0.05, *d* = 1.32, respectively). The results indicated a non-significant Condition × Time interaction for reduced motivation (*F* = 1.782, *p* = 0.115, $\eta_{p}^{2}$ = 0.106), reduced activation (*F* = 0.733, *p* = 0.628, $\eta_{p}^{2}$ = 0.047), and mental fatigue (*F* = 1.530, *p* = 0.183, $\eta_{p}^{2}$ = 0.093).

### Activation Deactivation Affective Check List

The repeated MANOVA revealed a Condition (3) × Time (5) interaction (*F* = 1.794, *p* < 0.01, $\eta_{p}^{2}$ = 0.108) for the ADACL subscales. The means and standard deviations of the ADACL subscales are presented in **Table 1**. As regards to tiredness, a Condition × Time interaction for tiredness (*F* = 2.257, *p* < 0.05, $\eta_{p}^{2}$ = 0.131), and a Time (*F* = 7.535, *p* < 0.001, $\eta_{p}^{2}$ = 0.201) but not Condition (*F* = 0.395, *p* = 0.677, $\eta_{p}^{2}$ = 0.026) main effect was observed. A change for tiredness appeared in the HAMB (*F* = 5.022, *p* < 0.01, $\eta_{p}^{2}$ = 0.334), with an increase from Pre to D7 (*p* < 0.05, *d*_z_ = 1.26). In the HBR, the tiredness changed (*F* = 5.351, *p* < 0.01, $\eta_{p}^{2}$ = 0.349) across intervention, and was higher on the D7 (*p* < 0.05, *d*_z_ = 1.09) and D14 (*p* < 0.01, *d*_z_ = 1.54) than in the Post measure. Finally, in the NBR, the changes over Time approached statistical significance (*F* = 2.250, *p* = 0.081, $\eta_{p}^{2}$ = 0.184).

**Table 1:** Descriptive statistics (mean ± SD) of the activation deactivation adjective check list (ADACL) subscales in the experimental conditions.

ADACL subscales	Condition	Pre *M* ±*SD*	D7 *M* ±*SD*	D14 *M* ±*SD*	D21 *M* ±*SD*	Post *M* ±*SD*
Energy	HAMB	9.00 ± 3.87	8.63 ± 3.11	8.82 ± 2.52	8.64 ± 1.96	9.27 ± 3.85
	HBR	10.27 ± 4.10	9.64 ± 4.15	9.82 ± 3.60	10.45 ± 4.25	11.27 ± 3.80
	NBR	8.27 ± 2.64	10.09 ± 3.81	10.09 ± 4.13	9.91 ± 4.37	10.09 ± 4.09
Tiredness	HAMB	12.09 ± 2.03	15.09 ± 2.34	12.64 ± 3.29	13.55 ± 2.62	13.27 ± 2.57
	HBR	12.82 ± 2.40	13.91 ± 2.51	15.27 ± 2.69	13.00 ± 2.90	11.73 ± 2.80
	NBR	11.55 ± 1.37	14.00 ± 2.14	13.09 ± 3.56	12.55 ± 2.88	12.09 ± 3.33
Tension	HAMB	11.82 ± 1.47	17.64 ± 2.50	13.82 ± 2.18	12.36 ± 2.01	13.36 ± 1.12
	HBR	11.18 ± 0.98	17.18 ± 3.52	17.18 ± 3.89	17.00 ± 3.07	12.64 ± 2.06
	NBR	12.64 ± 1.36	17.55 ± 3.59	15.55 ± 4.11	16.18 ± 3.89	12.64 ± 2.80
Calmness	HAMB	11.64 ± 3.04	11.36 ± 2.06	11.91 ± 2.07	11.64 ± 1.92	11.27 ± 1.35
	HBR	11.36 ± 2.46	11.27 ± 1.27	11.18 ± 1.94	11.82 ± 1.60	11.64 ± 2.62
	NBR	10.45 ± 1.44	11.09 ± 1.76	11.00 ± 1.90	11.63 ± 3.17	11.91 ± 2.47

*Pre, 2 days before the onset of each experimental condition; D7, 7th day of each experimental condition; D14, 14th day of each experimental condition; D21, 21st day of each experimental condition; Post, 1st day after each experimental condition; HAMB, hypoxic ambulatory condition; HBR, hypoxic bed rest condition; NBR, normoxic bed rest condition; TMD, total mood disturbance*.

The results indicate that for tension there was a significant Condition × Time interaction (*F* = 4.052, *p* < 0.001, $\eta_{p}^{2}$ = 0.213), as well as a Time (*F* = 32.313, *p* < 0.001, $\eta_{p}^{2}$ = 0.518) but not a Condition (*F* = 1.389, *p* = 0.265, $\eta_{p}^{2}$ = 0.085) main effect. The tension in the HAMB condition significantly changed (*F* = 20.147, *p* < 0.001, $\eta_{p}^{2}$ = 0.668), and was higher on D7 than in the Pre (*p* < 0.001, *d*_z_ = 1.81) intervention measure. Thereafter, tension decreased from D7 to the D14 (*p* < 0.01, *d*_z_ = 1.47), D21 (*p* < 0.001, *d*_z_ = 2.31), and the Post (*p* < 0.001, *d*_z_ = 1.95) measures. In the HBR condition, differences over time were also revealed (*F* = 19.630, *p* < 0.001, $\eta_{p}^{2}$ = 0.663). The tension increased from Pre to D7 (*p* < 0.01, *d*_z_ = 1.61), D14 (*p* < 0.01, *d*_z_ = 1.56), and D21 (*p* < 0.01, *d*_z_ = 1.74). Subsequently, tension decreased in the POST intervention measure compared to D7 (*p* < 0.01, *d*_z_ = 1.44), D14 (*p* < 0.01, *d*_z_ = 1.62), and D21 (*p* < 0.01, *d*_z_ = 1.37). Finally, in the NBR condition differences also appeared (*F* = 7.069, *p* < 0.001, $\eta_{p}^{2}$ = 0.414), as tension increased from Pre to D7 (*p* < 0.01, *d*_z_ = 1.46), and the increase on D21 compared to Pre measure approached statistical significance (*p* = 0.066, *d*_z_ = 1.03). Likewise, the decrease of tension from D7 to the Post approached statistical significance (*p* = 0.066, *d*_z_ = 1.03). The results did not indicate a significant Condition × Time interaction for energy (*F* = 0.627, *p* = 0.718, $\eta_{p}^{2}$ = 0.040) or calmness (*F* = 0.395, *p* = 0.922, $\eta_{p}^{2}$ = 0.026).

## Discussion

Our results suggest that combined hypoxia and BR-induced unloading (HBR) exert a detrimental effect on affective and fatigue responses as compared to the independent effects of hypoxia (HAMB) and BR (NBR). The affective and fatigue changes across the HAMB intervention were limited, suggesting that physical activity might attenuate the negative effects of both hypoxia and/or BR. Finally, the participants in the NBR intervention exhibited only moderate signs of fatigue.

### General Adaptation

Subjects adapted to the interventions without any significant health-related adverse effects. The profound systemic effect of hypoxia during both HAMB and HBR was clearly reflected in significantly reduced SpO_2_. The magnitude of the SpO_2_ reduction is also in line with other studies investigating exposures to similar simulated altitudes (Debevec et al., 2014b). Collectively, this indicates that both, hypoxia and BR provoked profound and expected physiological responses.

### The Effects of (In)activity

While the positive effects of exercise on affect and cognitive function have been reported previously (Hillman et al., 2008; Erickson et al., 2015), the exercise effects in hypoxic environments and/or in confinement have, to date, not been studied. In the present study a significant increase of the perception of general and physical fatigue was noted as an independent consequence of BR. In contrast to previous findings (Ishizaki et al., 2002, 2004; Liu et al., 2012; Stavrou et al., 2015) reporting no effect of BR *per se* on tension, our results indicate a significant increase in tension throughout the NBR intervention, and returned to baseline levels during the recovery period. The disparity of the present results compared to others (Liu et al., 2012) could, in part, be attributed to the differences in the BR duration, participants’ characteristics, and/or methodology applied.

The participants in both HBR and NBR displayed a significant increase in the general fatigue, while no differences were noted in the HAMB condition. Additionally, the participants in the HBR and NBR conditions, compared to HAMB, revealed higher perception of physical fatigue. A link between low levels of physical activity and fatigue has been shown previously (Chen, 1986; Kroenke et al., 1988). In particular, Vercoulen et al. (1996a,b) compared patients with chronic fatigue syndrome and suggested that the ones who were more physically active indicated lower levels of fatigue than those who were less active.

As mentioned earlier, fatigue is a multifactorial concept (Lewis and Wessely, 1992; Shen et al., 2006), which lacks a commonly accepted definition. Research on fatigue has focused on its relationship to a series of psychological and environmental factors, as well as, on the predispositions or the perpetuating factors of fatigue. Pennebaker (1982) and Rijk et al. (1999) noted the importance of environmental information/stimulation, suggesting that there is a “competition of cues” that might affect the psychological and behavioral responses. The quantity–quality model for understanding fatigue (Rijk et al., 1999) proposes the need for balance between the quantity and the quality of the cues to avoid/limit fatigue, highlighting the importance of psychological factors in fatigue modulation. In other words, high quantity of external stimulation (high “experienced overload”) and low quality of external stimulation (low “attractiveness of external stimulation”) are both related to higher fatigue levels, in a curvilinear manner (Pennebaker, 1982). Based on the above, in cases of high external information, the individual will experience negative emotions such as anxiety, whereas in situations of low quality of external stimulation, similar to those in the present study (BR, confinement), the environment is perceived as boring, which might lead to increased feeling of fatigue.

Comparison of affective responses between the active (HAMB) and inactive (HBR) interventions indicated that the HBR trial provoked the most negative affective responses (higher tension and tiredness) and perception of fatigue (higher general and physical fatigue), as their profile continuously deteriorated (D7, D14, and D21), even upon completion of the intervention. The fact that fatigue responses remained stable during the HAMB suggests that regular daily physical activity (two 30-min low-intensity exercise sessions) along with the other habitual activities performed during the HAMB compensated the psychological impairment noted in the HBR condition. The elevation of tension and tiredness in the beginning (D7) of the HAMB intervention was transient, returning to initial levels thereafter (D14, D21, Post), thus providing support to the potent effect of exercise (Stavrou et al., 2015) and participants’ social interaction to ameliorate psychological strain.

With regard to fatigue, limited research suggests that individuals’ participation in physical activity programs can ameliorate subjective feelings of fatigue (Puetz et al., 2006, 2008). A series of epidemiological studies have demonstrated that physical activity is positively related to low fatigue levels (Puetz and Herring, 2013). In line with this, Puetz (2006) supported the notion that physically active individuals, compared to sedentary ones, have a lower risk to experience fatigue. In addition to the epidemiological findings by which a cause-effect relationship cannot be determined, Puetz et al. (2006) conducted a meta-analysis of 70 randomized control trials showing that exercise can reduce fatigue in both healthy individuals and patients. In addition, compared to no exercise, the effects of low- and moderate-intensity exercise indicate a positive effect on the perception of fatigue in healthy individuals reporting persistent feelings of fatigue (Puetz et al., 2008). Their results lend further support to the notion that exercise has a positive effect on the fatigue level of healthy, sedentary individuals. The results of the present study also show potentiation of fatigue responses during both inactive (HBR and NBR) interventions, whereas, no such effect on fatigue was observed when the participants were habitually active (HAMB).

### The Effects of Hypoxia

The intervention combining BR and normobaric hypoxia (HBR) elicited the most negative affective state, and fatigue responses compared to both HAMB and NBR. HBR participants exhibited a significant increase in tiredness, tension, general fatigue, physical fatigue, which were higher than in HAMB and NBR. In line with previous studies (Stavrou et al., 2015, 2018), no effect was noted on the positive affect such as energy and calmness. These findings partly provide support for the counteracting effect of exercise against deterioration of affect and increase of fatigue in adverse environments (Stavrou et al., 2015). In particular, although the HAMB participants showed a significant increase in tension and tiredness only on D7 and D14 compared to the Pre measure, tension then decreased and returned to its initial level during the latter stages of the confinement, suggesting participants’ successful adaptation to the hypoxic environment. It is also noteworthy that perception of fatigue continuously increased during the HBR condition, indicating the highest values in the D21 and Post measures. Although causality cannot be argued based on the methodology of the current study, the increase of fatigue can be attributed to participants’ worsening of the affective state during the HBR. It can also be linked to the limited and low quality external stimulus that they received during the BR exposure, resulting in boredom and negative affect, which can lead to a feeling of fatigue (Pennebaker, 1982; Rijk et al., 1999). Nevertheless, our data seem supportive to the notion that high altitude exposure exerts an independent detrimental effect on psychological state and adaptation (Bahrke and Shukitthale, 1993; Stavrou et al., 2015).

The fact that the deterioration of fatigue and affect responses was not apparent in HAMB, confirms that BR-related inactivity *per se* may exert a detrimental effect. This, and the finding that hypoxia aggravates BR-induced negative affect and fatigue are in line with previous results (Stavrou et al., 2015). Fatigue, conceptualized as a mood in the current study, may constitute mental representations of physiological changes that characterize these emotions (Gibson et al., 2003). As such, the increased levels of fatigue in the hypoxic BR condition are indicators of physiological changes. As emotional responses are thought to be actuated through amygdala and the prefrontal cortex, a possible physiological explanation of the fatigue and affect deterioration might be a hypoxic effect on these brain regions. The prefrontal cortex seems to have a moderating effect exerting an inhibitory control on the amygdala during exposure to aversive stimuli, helping to regulate and shorten the duration of the negative affective responses (Davidson, 2002). Davidson et al. (2000) note that when the inhibition is absent “*the amygdala remains unchecked and continues to [be] activated.*” However, it is difficult to support the aspect that the “*fatigue is localized [in a] single area of the brain*” as a variety of psychological responses (i.e., motivation, anger, and depression) and cognitive functioning is linked to fatigue (Gibson et al., 2003) responses in which various brain regions are involved (Bassin et al., 1999). Based on this, it would seem worthwhile for future studies to also examine alterations in brain activity through electrophysiological methods that can provide important information in the interpretation of individual psychological responses and adaptation in hypoxic environment (Ozaki et al., 1995; Wilson et al., 2009; Richardson et al., 2011). Furthermore, polysomnography measurements could also provide a valuable approach for future investigations into the combined and separate effects of hypoxia and inactivity on physical and mental fatigue.

### Implications and Methodological Considerations

Whether the levels of hypoxia and unloading/reduced physical activity during future long term missions to the Moon and Mars will be as severe as to alter affect and perceived fatigue sensation remains to be established. It has to be noted that astronauts regularly and frequently perform physical exercise. Notwithstanding, our findings clearly indicate that the addition of hypoxia might negatively impact both affect and fatigue responses and should be taken into account. As noted during the HAMB, exercise during future space habitation will not only be beneficial for maintaining physiological well-being of the mission participants, but will also provide beneficial effects on fatigue and psychological resistance. This should be considered especially, given that optimal cognitive function, psychological responses, and social interactions have been identified as key parameters that determine successful completion of space missions (Manzey, 2004; Kanas et al., 2009).

In addition, future investigations should also focus on the notable inter- and intra-individual variability of the participants’ affect and fatigue responses, and also relate them to the participants’ personality characteristics as these are known to be associated to subjective emotional experiences (Watson, 1988). Further investigations are also warranted to better understand the underlying mechanisms of perceived fatigue and to define its role in psychological responses in adverse environments. Finally, given that normobaric hypoxia was used as a surrogate of hypobaric hypoxic exposure, the potential differential affect and fatigue responses to either normobaric and/or hypobaric hypoxia should also be further explored.

## Conclusion

The present findings clearly show that hypoxia deteriorates individuals’ psychological profile and enhances fatigue responses in bed rest participants, but not in active, ambulatory participants. This further suggests that the hypoxic effect is transient, since it is not sustained once the hypoxic stimulus is removed. However, the effect of hypoxia and/or bed rest on fatigue responses seems to be different, as general and physical fatigue gradually increased throughout the intervention, remaining elevated even after the cessation of the HBR exposure. On the other hand, it seems that even low physical activity levels are sufficient to negate the fatigue responses and negative affect induced by a hypoxic environment.

## Author Contributions

IM and OE initiated the overall research project and designed the study. TD, IM, and OE were involved in the measurements and data collection. All authors were involved in the analysis and interpretation of the results, as well as in the preparation of the manuscript.

## Conflict of Interest Statement

The authors declare that the research was conducted in the absence of any commercial or financial relationships that could be construed as a potential conflict of interest.
